# GCaMP fiber photometry reveals mechanistic insights into focused ultrasound neuromodulation in acute seizures

**DOI:** 10.1038/s44384-026-00057-6

**Published:** 2026-09-02

**Authors:** Chen-Syuan Huang, Yi-Chun Yeh, Po-Chun Chu, Yi-Tse Hsiao, Hsiang-Yu Yu, Robert S. Fisher, Hao-Li Liu

**Affiliations:** 1 Department of Electrical Engineering, National Taiwan University, Taipei, Taiwan; 2 Graduate Institute of Biomedical Electronics and Bioinformatics, National Taiwan University, Taipei, Taiwan; 3 Department of Veterinary Medicine, School of Veterinary Medicine, National Taiwan University, Taipei, Taiwan; 4 Department of Neurology, Taipei Veteran General Hospital, Taipei, Taiwan; 5 School of Medicine, National Yang Ming Chiao Tung University, Taipei, Taiwan; 6 Department of Neurology, Stanford University School of Medicine, Palo Alto, CA USA

**Keywords:** Neurology, Neuroscience

## Abstract

Generalized seizures represent a major clinical challenge in epilepsy, often resistant to pharmacological treatment and associated with significant morbidity. While low-intensity focused ultrasound (FUS) has emerged as a promising noninvasive neuromodulation strategy to suppress epileptiform activity, the underlying calcium mechanism remains poorly understood. This study, utilizing simultaneous hippocampal GCaMP fiber photometry and electrocorticography (ECoG) in acute pentylenetetrazol-induced seizure mouse models, investigated whether FUS stimulation could modulate intracellular calcium dynamics and network excitability. FUS was applied to the hippocampus with a mechanical index of 0.2, a duty cycle of 7.5%, and a total duration of 10 min. Our findings demonstrated a strong correlation between epileptic calcium transients and ECoG spikes, and FUS significantly attenuated both modalities for up to 40 min. Immunofluorescence analyses in the dentate gyrus revealed decreased c-Fos expression co-localized with both NMDAR2B and GAD65/67 after FUS treatment, indicating an overall reduction in both excitatory and inhibitory synaptic transmission. These results suggest that low-intensity FUS exerts calcium-dependent anti-seizure effects by modulating the excitatory-inhibitory balance. By providing real-time insights into neural network excitability, GCaMP fiber photometry complements traditional electrophysiology and serves as a potent mechanistic surrogate for assessing therapeutic efficacy, potentially guiding future closed-loop neuromodulation strategies.

## Introduction

Epilepsy is a common neurological disorder marked by recurrent seizures from abnormal brain activity, affecting over 60 million people worldwide^1^. About one-third develop drug-resistant epilepsy, underscoring the need for new noninvasive treatments for those ineligible for surgery^2^. Clinically, the International League Against Epilepsy classifies seizures as focal (from localized regions) or generalized (widespread, synchronous activity across both hemispheres, including thalamocortical circuits)^3–5^.

A key pathological feature of epilepsy is disrupted calcium homeostasis^6^. Calcium ions (Ca²⁺) act as essential second messengers, significantly influencing neuronal excitability, synaptic transmission, and activity-dependent gene expression^7^. Abnormal calcium signaling, particularly via N-methyl-D-aspartate receptors (NMDARs)-mediated influx, contributes to hyperexcitability and ictogenesis^3,5^. Dysregulated or excessive calcium influx, via NMDARs and other receptors, can result in neuronal hyperexcitability and aberrant network synchronization, key features of ictogenesis^8^. In the pentylenetetrazol (PTZ) seizure model, which is used to study generalized seizures, PTZ antagonizes GABA_A_ receptors, reducing chloride ions influx and diminishing inhibitory neurotransmission^9,10^. The disinhibition results in membrane depolarization and indirectly activates various calcium channels, including NMDARs, TRPV1 and voltage-gated calcium channels (VGCCs)^11–13^. Increased calcium influx promotes excitatory neurotransmitter release and ultimately triggers epileptiform discharges^14^.

Recent advances have demonstrated that low-intensity focused ultrasound (FUS) stimulation can precisely target deep brain structures and exert neuromodulatory effects via mechanical interactions with neuronal membranes, reducing seizures in animal models^15,16^. FUS modulates neuronal activity through interactions with various mechanosensitive ion channels, including Ca^2+^ channels, such as Piezo1 and TRPA1^17–19^, modulating calcium influx and influencing membrane potentials, neuronal excitability, and synaptic transmission^20–22^. Previous studies have reported that FUS effectively suppresses chemically-induced epileptiform activity by modulating GABAergic neuronal activity and enhancing inhibitory neurotransmission^23–25^. Specifically, FUS is postulated to exert seizure-suppressive effects by driving calcium influx in inhibitory interneurons, thereby reducing the activity of excitatory pyramidal cells^26^. However, the detailed cellular and molecular mechanisms underlying the antiseizure effects of FUS remain incompletely understood and warrant further investigation.

For epilepsy monitoring, electroencephalography (EEG) and electrocorticography (ECoG) are widely employed preclinical and clinical tools for real-time seizure detection, offering high temporal resolution suitable for evaluating seizure suppression after FUS treatment^27–29^. However, these electrophysiological methods have limitations, notably their inability to directly capture intracellular ion signaling^30^. GCaMP fiber photometry recording, using genetically encoded calcium indicators, provides an alternative approach that allows real-time monitoring of neuronal calcium dynamics at the population level^31,32^. This technique complements traditional electrophysiology by offering critical insights into calcium-mediated signaling with high spatial specificity, enabling the monitoring of deep-brain structure, specifically the hippocampus, that remain challenging to isolate using surface EEG or ECoG due to volume conduction.

Disrupted calcium homeostasis, particularly excessive calcium influx through receptors, such as NMDARs, is widely implicated in the pathophysiology of epilepsy, contributing to neuronal hyperexcitability and aberrant network synchronization. Fiber photometry, a technique extensively employed in preclinical neuroscience research using genetically encoded calcium indicators, such as GCaMP, enables real-time monitoring of population-level calcium dynamics in vivo. Despite its proven utility, GCaMP fiber photometry has not yet been applied to investigate the neuromodulatory effects of FUS in the context of epilepsy, particularly in relation to calcium signaling. We hypothesize that GCaMP fiber photometry recording can address this critical gap by capturing real-time calcium dynamics and network excitability in a preclinical epilepsy model. This approach offers strong potential to elucidate calcium-dependent mechanisms underlying FUS-mediated seizure suppression and to advance our understanding of noninvasive neuromodulation strategies for drug-resistant epilepsy.

The primary objective of this study is to evaluate whether fiber photometry-based calcium signals can serve as a reliable surrogate for both neuronal hyperexcitability and the therapeutic efficacy of FUS neuromodulation in epilepsy. By enabling real-time monitoring of network-level excitability in vivo, GCaMP fiber photometry provides a unique physiological indication that complements traditional electrophysiology. We aim to validate the correlation between calcium dynamics and conventional ECoG to establish this multimodal approach as a robust preclinical platform, which is critical for facilitating the optimization of acoustic parameters and the future translation of safe and effective clinical protocols. To further delineate the molecular and cellular mechanisms underlying the neuromodulatory effects of FUS, particularly its influence on calcium-related neural activity, complementary immunofluorescence analyses targeting activity-dependent markers c-Fos, the NMDAR2B subunit (implicated in excitatory calcium influx), and glutamate decarboxylase 65/67 (GAD65/67; critical for GABA synthesis and inhibitory signaling). Through this multimodal strategy, the study investigates how FUS alters the balance of excitatory and inhibitory neurotransmission at both functional and molecular levels. Together, we aim to deepen mechanistic insight into FUS-mediated seizure suppression and support the advancement of noninvasive, circuit-specific interventions for drug-resistant epilepsy.

## Results

### Calcium signaling as an effective surrogate for detecting epileptiform activity and evaluating FUS-mediated suppression

**Fig. 1:**
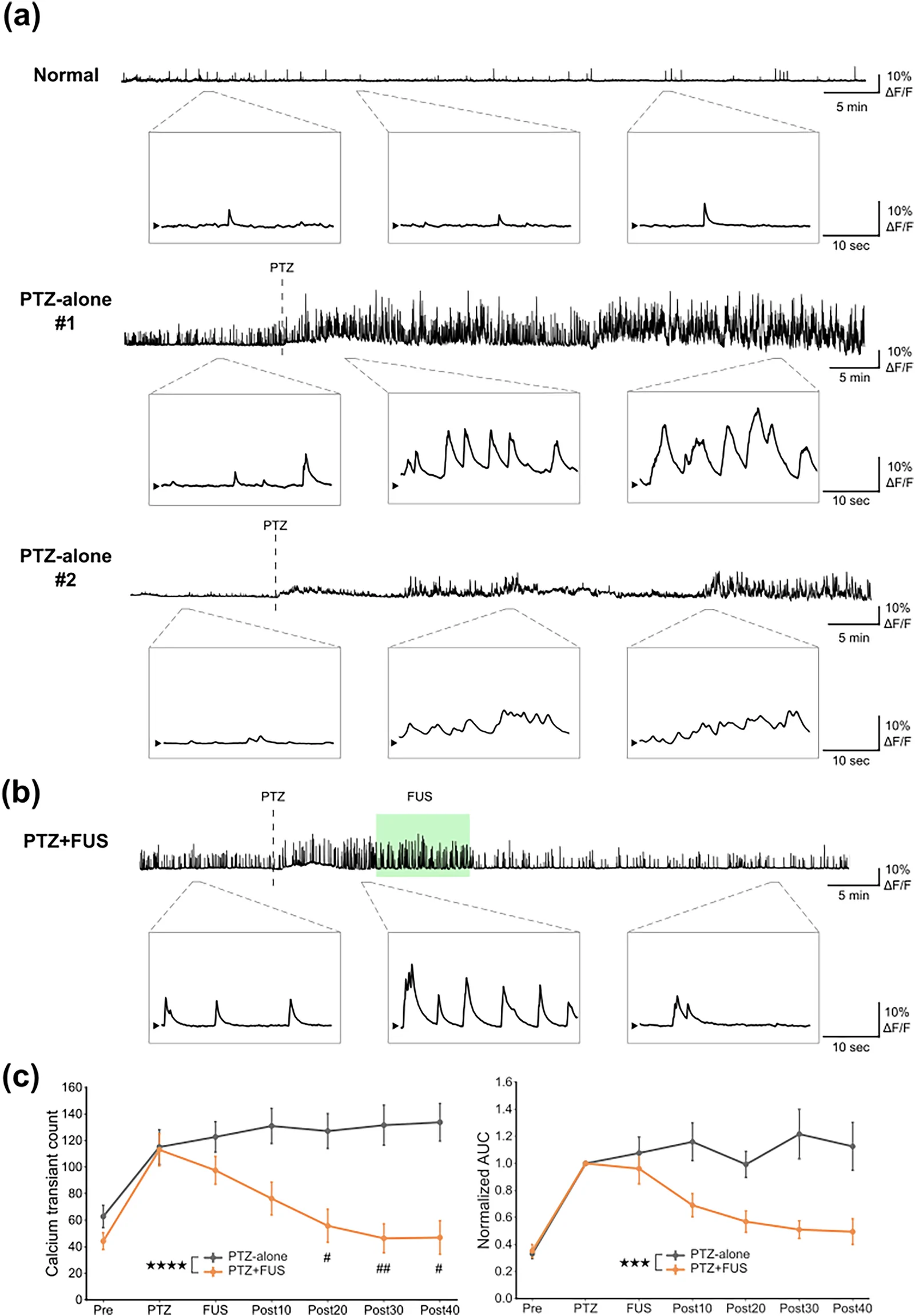
FUS-mediated suppression of epileptiform calcium transients in a PTZ-induced seizure model. **a** Representative GCaMP fluorescence traces from normal and PTZ-alone mice. Insets show expanded views of calcium transients, with stable low-level activity in controls and elevated amplitude/frequency following PTZ. **b** Representative trace from a PTZ + FUS mouse shows a marked reduction in calcium activity during and after FUS (green shading). **c** Quantification of calcium transient count (left) and normalized area under the curve (AUC, right) across time points. FUS significantly reduced both metrics compared to PTZ-alone (*n* = 7 for PTZ-alone, *n* = 10 for PTZ + FUS). Statistical comparisons were made using linear mixed model (^★★★^*p* < 0.001, ^★★★★^*p* < 0.0001) and post hoc (^#^*p* < 0.05, ^##^*p* < 0.01). Data are shown as mean ± SEM.

A PTZ-induced acute seizure model was used to assess whether GCaMP fiber photometry signals reliably reflect epileptiform activity. FUS targeted the hippocampus to modulate calcium signals in the PTZ + FUS group throughout the experimental time course, assessing FUS effects on epileptic activity. In the normal group, calcium signals remained stable, showing low amplitude and frequency (Fig. 1a). In contrast, PTZ injection induced an increase in both the frequency and amplitude of calcium transients, along with sustained calcium accumulation (Fig. 1a). Following PTZ induction, FUS stimulation effectively suppressed these aberrant responses, with both the amplitude and frequency of calcium signals reduced during and after sonication (Fig. 1b).

Calcium transient count in the PTZ + FUS group decreased from 113 ± 12 events (PTZ period) to 76 ± 12 at 10 min post-FUS. Linear mixed model analysis revealed a significant main effect of group (*F*_(6, 86.14)_ = 6.15, *p* < 0.0001), indicating an overall suppression through 40 min after treatment. Specifically, calcium transients were significantly reduced starting 20 min after FUS stimulation (53 ± 16 at Post20, 40 ± 13 at Post30 and 41 ± 13 at Post40, post hoc *p* < 0.05; Fig. 1c left). Similarly, normalized AUC decreased from 1.0 ± 0.0 during the PTZ period to 0.69 ± 0.09 immediately after FUS, showing a significant overall reduction across all post-FUS periods (*F*_(6, 74.00)_ = 4.73, *p* < 0.001 vs. PTZ-alone group; Fig. 1c right); normalized transient amplitude also exhibited a similar trend, dropping to 0.73 ± 0.06 10 min after FUS, and showed a sustain suppression (*F*_(6, 88.06)_ = 2.15, *p* = 0.0555 vs. PTZ-alone group; Fig. S1), suggesting that FUS effectively modulates both the magnitude and frequency of calcium dynamics associated with epileptiform activity.

### Simultaneous cortical and hippocampal recordings demonstrating FUS disruption of generalized epileptiform activity

**Fig. 2:**
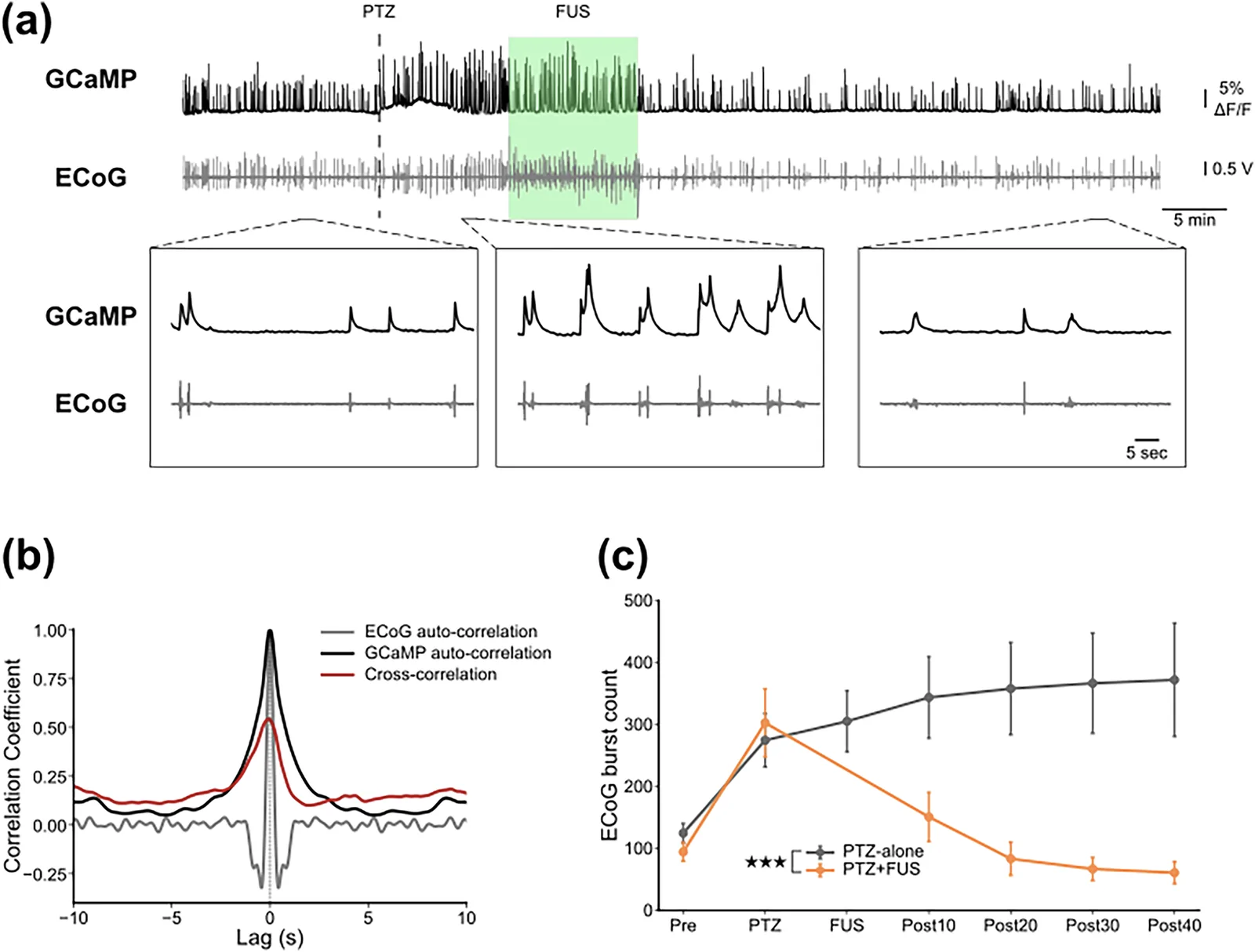
FUS-mediated attenuation of epileptiform activity in GCaMP fluorescence and ECoG. **a** Representative GCaMP fiber photometry and ECoG traces show elevated calcium and spike activity after PTZ injection, which is suppressed during and after FUS (green shading). Insets illustrate detailed calcium and ECoG signal reduction post-FUS. **b** Representative cross-correlation analysis reveals strong temporal alignment between calcium and ECoG signals. **c** Quantification shows that ECoG spike counts significantly decreased post-FUS compared to PTZ controls (*n* = 7 for PTZ-alone, *n* = 5 for PTZ + FUS; linear mixed model; ^★★★^*p* < 0.001). Data shown as mean ± SEM.

To examine whether FUS stimulation can disrupt generalized epileptiform activity across brain regions, cortical ECoG and hippocampal GCaMP signals were recorded during PTZ-induced seizures. During seizure episodes, ECoG and calcium signals exhibited strong temporal synchrony, confirming coordinated activation between cortical and hippocampal networks (Fig. 2a). Cross-correlation analyses between ECoG and calcium signals for each recording were computed, and the calcium signal was delayed with respect to the ECoG signal by 0.03 ± 0.05 s on average (Fig. 2b). This minor delay is specific to high-amplitude burst discharges and is primarily attributed to hardware synchronization constraints between independent acquisition systems rather than biological kinetics, confirms that the two modalities are highly synchronous relative to the minute-scale FUS intervention. Furthermore, regression analysis revealed a robust linear correlation between ECoG spike and GCaMP transient counts (*R*^2^ = 0.73, *p* < 0.001; Fig. S2). These examinations validate tight coupling between the two modalities and support GCaMP calcium fluorescence as a reliable surrogate of seizure dynamics.

Following FUS treatment, ECoG spike count in the PTZ + FUS group decreased from 303 ± 55 spikes (PTZ period) to 151 ± 39 spikes at 10 min post-FUS, and declined significantly throughout 40 min after treatment (*F*_(5, 50)_ = 5.02, *p* < 0.001 vs. PTZ-alone group; Fig. 2c). These findings suggest that FUS stimulation effectively disrupts synchronized epileptiform activity in hippocampus and cortex, resulting in a delayed but sustained off-line neuromodulatory effect.

### Spectral analysis of cortical ECoG confirming delayed and sustained anti-epileptic efficacy of FUS

**Fig. 3:**
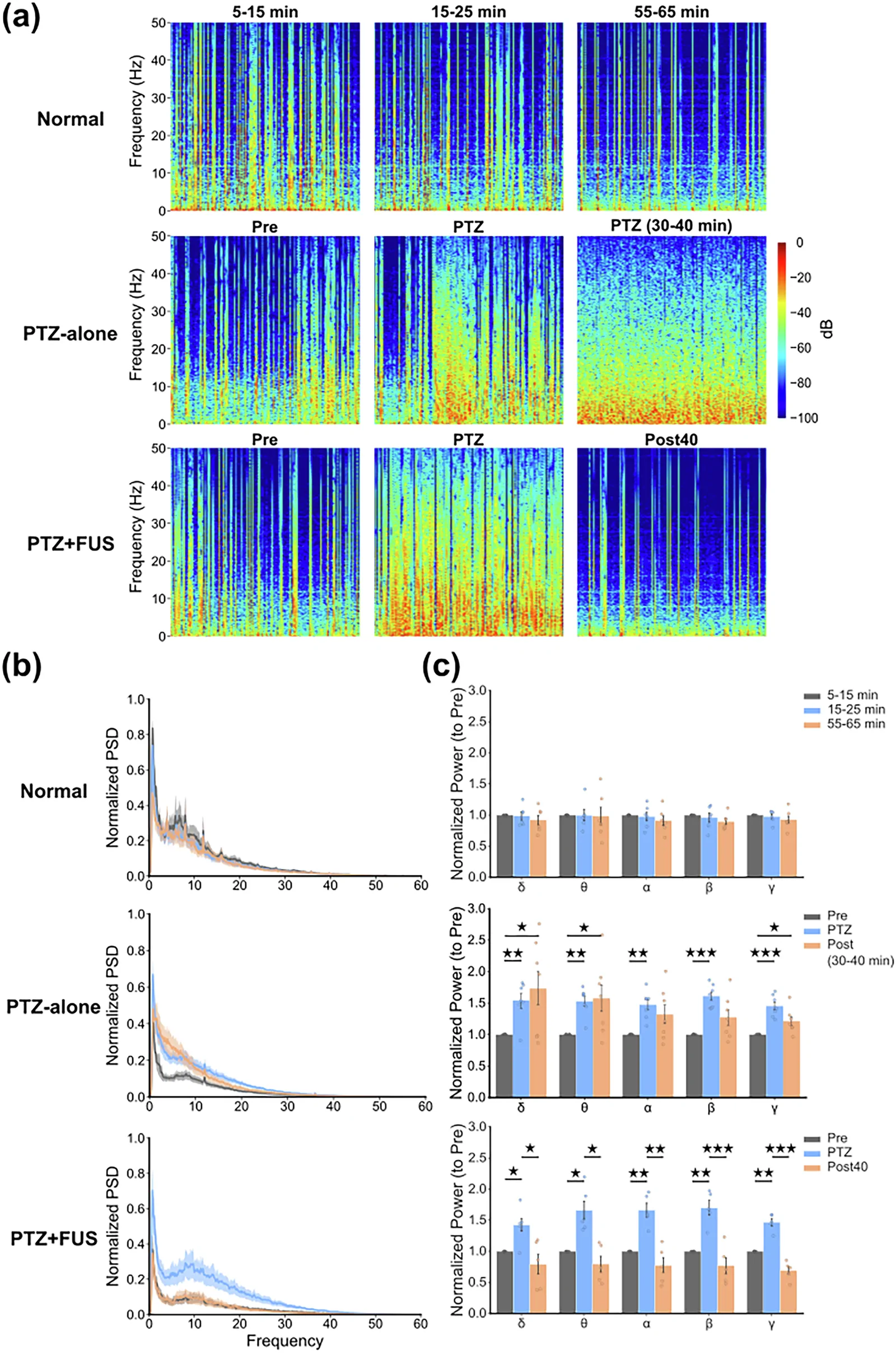
Spectral analysis of ECoG showing FUS-induced attenuation of epileptiform activity. **a** Representative spectrograms show dynamic frequency changes across three time periods: baseline (Pre), during PTZ-induced seizures (PTZ), and the final recording epoch (55–65 min for the normal group, Post30–40 min for the PTZ-alone group and Post40 for the PTZ + FUS group). In PTZ-alone mice (middle), PTZ increased spectral power across multiple frequency bands. In contrast, FUS-treated mice (bottom) exhibited reductions in spectral power following stimulation. **b** Normalized PSD plots for normal, PTZ-alone, and PTZ + FUS groups. PTZ administration elevated broadband activity, particularly in the low-frequency range, which was significantly suppressed after FUS. **c** Group-level quantification of normalized spectral power across delta (1–4 Hz), theta (4–8 Hz), alpha (8–13 Hz), beta (13–30 Hz), and gamma (30–60 Hz) bands. FUS significantly reduced PTZ-induced increases across all bands. (*n* = 6 for normal, *n* = 7 for PTZ-alone, *n* = 5 for PTZ + FUS; two-tailed one-sample t-test and paired t-test; ^★^*p* < 0.05, ^★★^*p* < 0.01, and ^★★★^*p* < 0.001). Data are *p*resented as mean ± SEM.

Spectral analysis of ECoG signals was conducted to quantify the effects of FUS stimulation on cortical activity (Fig. 3a). PTZ administration induced a broad increase in cortical spectral power across delta (1–4 Hz), theta (4–8 Hz), alpha (8–13 Hz), beta (13–30 Hz), and gamma (30–60 Hz) bands, consistent with the electrophysiological signature of generalized seizures. Consistent with the cortical ECoG results, GCaMP spectrograms also showed increased spectral power in the delta (1–4 Hz) range following PTZ administration (Fig. S3), indicating network-level hyperexcitability.

No immediate reduction in spectral power was observed following FUS stimulation. However, power spectral density (PSD) analysis revealed a clear decrease in power beginning around 10 min after sonication, which persisted throughout the 40-min post-stimulation period (Fig. 3b). This delayed suppression pattern was consistent with the changes seen in time-frequency spectrograms (Fig. 3a).

Group-level spectral analysis revealed distinct long-term outcomes between groups. In the PTZ-alone group (*n* = 7), while normalized spectral powers of all bands were significantly elevated early after PTZ administration, the gamma band remained significantly increased at 40 min post-PTZ (1.22 ± 0.08-fold; *p* < 0.05 vs. Pre), reflecting persistent cortical hyperexcitability. In contrast, the PTZ + FUS group (*n* = 5) exhibited a broadband power suppression 40 min after treatment, with the most pronounced and significant reduction in the high-frequency beta (0.77 ± 0.14-fold) and gamma bands (0.69 ± 0.07-fold; *p* < 0.001; Fig. 3c), indicating that FUS induces a delayed but sustained suppression of PTZ-evoked cortical oscillations. This network-level suppression may reflect calcium-dependent or circuit-specific neuromodulatory effects of low-intensity FUS.

### Immunofluorescent analysis of cellular and molecular mechanisms underlying FUS-mediated seizure suppression

**Fig. 4:**
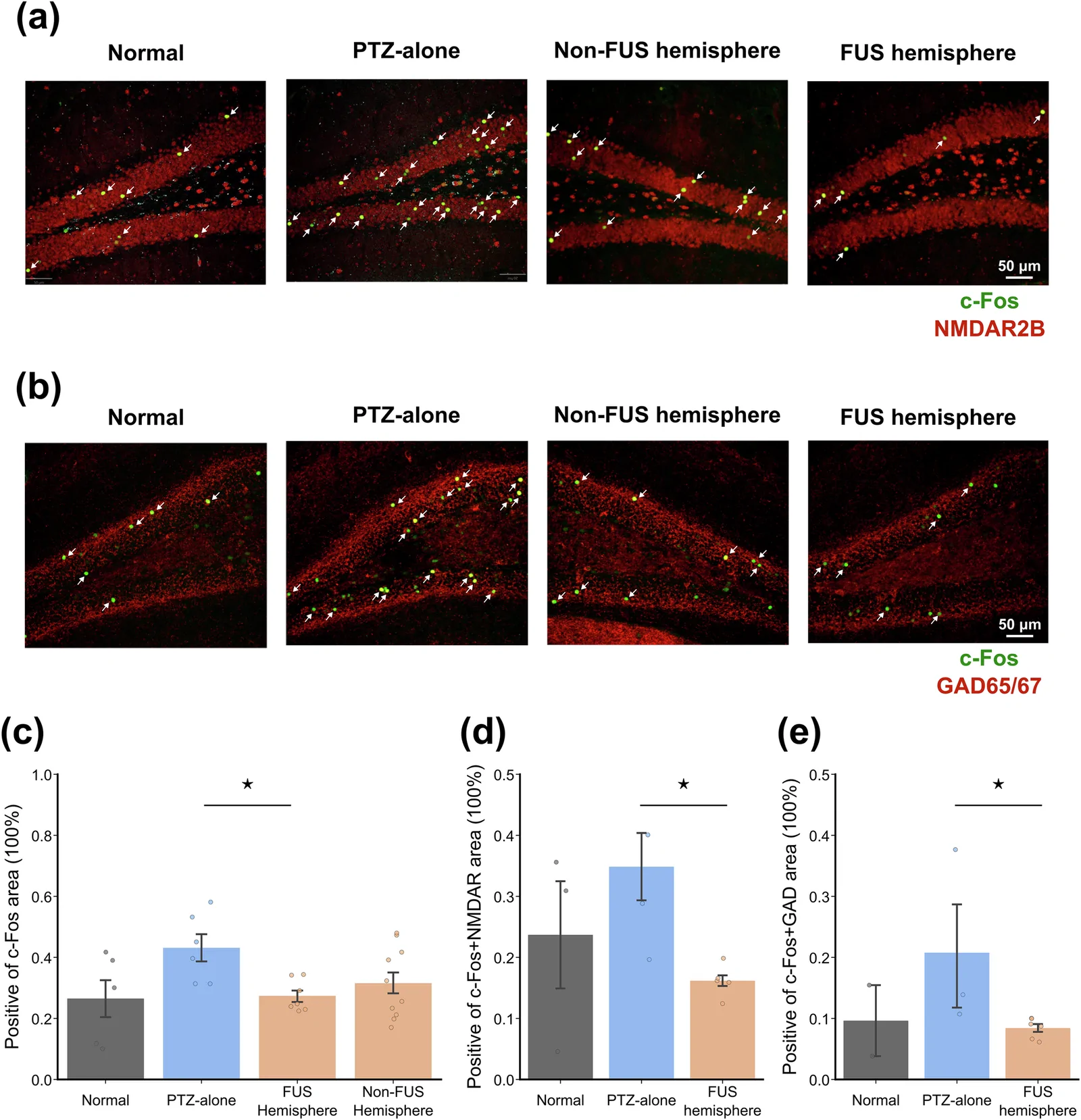
FUS suppression of neuronal hyperactivity and receptor co-expression in the dentate gyrus. Representative immunofluorescence images of the dentate gyrus showing the co-staining of neuronal activation marker c-Fos with the NMDA receptor subunit NMDAR2B: (**a**) and glutamic acid decarboxylase 65/67 (**b**). Images compare with Normal, PTZ-alone, and PTZ + FUS groups, with both the FUS-treated and contralateral (non-FUS) hemispheres included. White arrows indicate c-Fos or co-labeled c-Fos⁺/NMDAR2B⁺ cells. Scale bar = 50 μm. Quantification of the percentage area occupied by c-Fos⁺: (**c**), c-Fos⁺/NMDAR2B⁺ co-localized (**d**) and c-Fos⁺/GAD65 + 67 co-localized (**e**) signals within regions of interest (ROIs). FUS treatment significantly reduced the co-labeled area compared to the PTZ-alone group. (*n* = 5 for Normal group, *n* = 6 for PTZ-alone, *n* = 7 for FUS hemisphere from PTZ + FUS; two-tailed Wilcoxon rank-sum test; ^★^*p* < 0.05). Data are presented as mean ± SEM.

To examine the cellular mechanisms underlying FUS-mediated suppression of neuronal hyperactivity, immunofluorescent staining was performed in the hippocampal dentate gyrus (DG). Compared to the low basal expression in Normal animals, c-Fos expression, a marker of neuronal activation, was markedly increased in the PTZ-alone group. This PTZ-induced activation was visibly reduced in the FUS hemisphere of the PTZ + FUS group. (Fig. 4a, b). Quantitative analysis confirmed the c-Fos positive area percentage increased from 0.27 ± 0.15% in the normal group to 0.43 ± 0.11% in the PTZ-alone group. FUS treatment applied after PTZ administration significantly attenuated this neuronal activation, reducing the c-Fos positive area percentage to 0.27 ± 0.05% in the FUS hemisphere (*p* < 0.05 vs. PTZ-alone group; Fig. 4c). To further characterize the neuronal mechanisms involved, we measured the c-Fos signal co-localization between the NMDA receptor subunit NMDAR2B and GAD65/67. We found that FUS treatment also led to a significant reduction in the c-Fos–NMDAR co-localized signal area, from 0.35 ± 0.14% in the PTZ-alone group to 0.16 ± 0.03% in the FUS hemisphere of the PTZ + FUS group (*p* < 0.05), and the c-Fos-GAD co-localized area were as well reduced from 0.21 ± 0.15% in the PTZ-alone group to 0.08 ± 0.02% in the FUS hemisphere of the PTZ + FUS group (*p* < 0.05; Fig. 4d, e) Collectively, these data indicate that FUS effectively attenuating seizure-induced neuronal hyperactivity by suppressing the activity of both excitatory and inhibitory neuronal populations.

## Discussion

This study provides compelling functional and mechanistic evidence that FUS stimulation effectively suppresses PTZ-induced epileptiform activity and the hippocampal calcium signal, underscoring a possible important role of neuronal calcium dysregulation in epilepsy pathophysiology. Utilizing a multimodal approach including GCaMP fiber photometry, ECoG and immunofluorescence, we demonstrated that FUS reduces neuronal hyperexcitability in association with calcium-dependent mechanisms, evidenced by reduced pathological calcium and decreased NMDAR2B expression. A pivotal advancement is the successful application of GCaMP fiber photometry as a novel, real-time physiological surrogate for seizure dynamics and FUS therapeutic efficacy, establishing a critical foundation for developing sophisticated, adaptive closed-loop FUS neuromodulation systems. These findings underscore the significant therapeutic potential of low-intensity FUS as a safe, effective, and noninvasive strategy for treating drug-resistant epilepsy, strongly supporting the need for further investigation in chronic models and clinical settings.

Building upon the understanding that neuronal calcium dysregulation is central to epilepsy pathophysiology, this study supports the value of GCaMP fiber photometry calcium recording to directly detect and monitor PTZ-induced pathological calcium disturbances and their suppression following FUS stimulation. Through multimodal assessments, including real-time calcium recording, ECoG recordings, and immunofluorescence staining, we consistently demonstrated that FUS stimulation reduces neuronal hyperexcitability and suppresses epileptiform discharges, while modulating activity-related molecular markers. Our findings suggest that FUS neuromodulation may be mediated in part through a calcium-related process, reflected in the significant reduction of pathological intracellular calcium activity. Crucially, GCaMP provided spatial confirmation of target engagement within deep-brain structures that remains challenging for surface ECoG. Mechanistic insights further showed that FUS treatment influenced the expression of NMDAR2B (one of the key mediators of calcium influx^33^) and GAD65 + 67 (involved in GABA synthesis), indicating a potential role in restoring the excitatory/inhibitory balance disrupted in epilepsy. Moreover, the observed delayed yet sustained reductions in both calcium transients and ECoG spikes highlight the potential for FUS to induce lasting network desynchronization. This offline suppressive effect aligned with prior observations^26^. These results underscore the significant therapeutic potential of FUS for epilepsy by effectively targeting underlying calcium dysregulation and successfully establish GCaMP fiber photometry as a robust preclinical tool for mechanistic validation and FUS parameter optimization, which is critical for advancing the development of future closed-loop FUS neuromodulation systems for drug-resistant epilepsy.

In this study, the hippocampal area was chosen as the primary target for FUS to suppress PTZ-induced epileptiform activity. PTZ-induced generalized seizures are characterized by abnormal, widespread, and highly synchronous neuronal discharges involving multiple brain regions^34^. Although the thalamus is a common target for seizure suppression in generalized epilepsy models, the hippocampus, particularly the DG, which acts as a critical gating structure, plays a crucial role in seizure initiation and propagation^35^. Several studies have demonstrated that low-intensity FUS can disrupt aberrant neural oscillations and coupling in the hippocampus, thereby attenuating epileptiform activity^36–38^. Based on the above viewpoints, it’s a reasonable strategy to target the hippocampus with FUS in an effort to suppress epileptiform activity, particularly with focal onset seizure. With both focal and generalized onset seizures, sonication of the thalamus might beneficially disrupt seizure propagation networks.

Although the FUS was targeted at the unilateral hippocampal DG, the acoustic beam actually traversed the overlying cerebral cortex. The cortex is critically involved in initiating and spreading various types of seizures, including generalized seizure^39^. Moreover, low-intensity FUS has also been reported capable of reducing connectivity within epileptic circuits and the overall brain network^40^. The unintended yet unavoidable exposure may have contributed to neuromodulatory effects in the cortical regions as well. The observed suppressive effect on hippocampal calcium transients and cortical spike activity on the contralateral hemisphere suggests that FUS has a potential cross-hemispheric neuromodulatory effect, possibly through indirect network pathways or transcallosal projection mechanisms^37,41^. These findings support the idea that targeting FUS to both the cerebral cortex and the hippocampus can effectively suppress the propagation of epileptiform activity across an interconnected epileptic network.

Disrupted NMDA receptor NR2B signaling and impaired GABAergic inhibition are core features of epileptic networks^42,43^. In this study, we specifically focused on the DG for histological quantification. The DG acts as the gatekeeper of the hippocampus, filtering excitatory input from the entorhinal cortex^44^. Breakdown of this gate is a hallmark of temporal lobe epilepsy, allowing pathological excitability to propagate into the downstream hippocampal-cortical loop^45^. Therefore, molecular changes in the DG serve as sensitive indicators of both seizure severity and the restorative effects of FUS neuromodulation. We found that PTZ-induced epileptiform activity elevated hippocampal expression of c-Fos and NMDAR2B, indicating excessive excitatory drive. Concurrently, we observed an increase in the GAD65/67 expression in the PTZ-alone group. This likely represents a compensatory upregulation of inhibitory mechanisms in response to intense seizure activity, a phenomenon supported by previous studies that hippocampal interneurons upregulate GAD expression in an attempt to restore homeostatic balance during epileptogenesis^46,47^. Following FUS exposure, the levels of excitatory markers were significantly reduced, aligning with prior studies showing that low-intensity ultrasound can attenuate neuronal excitability^26,48^. Additionally, FUS reversed the elevation of GAD65/67. Rather than indicating the loss of inhibition tone, this reduction suggests that FUS treatment effectively suppresses pathological network hyperactivity, thereby removing the compensatory GABA synthesis and restoring the network to a lower-activity state.

GCaMP fiber photometry recording results show that pathological calcium surges were suppressed following FUS exposure, while the reduction in NMDAR2B expression emerged later in post-hoc histology. Previous in vivo studies have shown that parvalbumin-positive (PV⁺) interneurons are preferentially sensitive to low-PRF ultrasound, responding within milliseconds and at lower thresholds than excitatory pyramidal neurons^21,49,50^. These findings suggest that FUS may first preferentially activate PV⁺ interneurons, resulting in rapid, network-wide inhibition that acutely suppresses pathological calcium waves. Over time, this sustained inhibitory input could reduce pyramidal cell excitability and subsequently downregulate NMDAR2B expression, contributing to the long-term restoration of excitatory/inhibitory balance. Future studies incorporating dual-color photometry or cell-type-specific calcium activities will be crucial to directly verify this timing-dependent mechanism.

While EEG and ECoG provide real-time electrophysiological biomarkers for seizure detection, they do not directly capture intracellular signaling changes, such as calcium dynamics. In this study, we introduce GCaMP fiber photometry as a novel, population-level indicator for detecting epileptic activity and assessing FUS-mediated neuromodulation. Calcium signaling plays a central role in regulating neuronal excitability and synaptic plasticity, mediated largely through NMDARs and VGCCs^7,8^. Our results demonstrated that PTZ-induced epileptic signals were associated with prominent calcium surges, which were significantly attenuated by low-intensity FUS stimulation.

Although GCaMP fiber photometry lacks single-cell resolution, it provides robust measurements of dynamic calcium activity across neuronal populations and complements traditional electrophysiological recordings^51,52^. Importantly, while AAV transfection poses barriers to direct human applications, fiber photometry serves as a powerful preclinical platform for cellular mechanism investigation and FUS parameters validation. Calcium recordings reflect synchronized neuronal output and reveal the intracellular state of the network.

This modality allows precise, high-fidelity optimization of FUS parameters (e.g., MI, PRF, or duty cycle) that cannot be resolved by EEG/ECoG alone. Once these optimal parameters are validated via calcium dynamics in animal models, they can be translated to clinical protocols where patient monitoring relies on standard noninvasive EEG, paving the way to the development of closed-loop FUS neuromodulation system^53^. Adaptive FUS protocols may enable real-time interventions that respond to evolving seizure activity^21^, representing a critical step from mechanistic studies toward safe and precision clinical translation in drug-resistant epilepsy.

Recent studies have shown that various ultrasound therapy designs can suppress generalized epileptic activity with differing degrees of efficacy. For instance, Chen et al. demonstrated an 88.5% reduction in epileptic spikes by using a MI of 0.75 and a 30% duty cycle (DC)^23^. However, this high-intensity protocol raised concerns about thermal effects on superficial cortical tissues due to increased acoustic energy deposition^27^. As a safer alternative, other approaches have employed repeated or prolonged sonication at lower energy levels, achieving cumulative anti-epileptic effects while minimizing heating risks^27,54,55^. To further mitigate transcranial heating, recent work has incorporated inter-stimulus intervals (ISI), allowing time for thermal dissipation between sonications while maintaining therapeutic efficacy^56^.

In this study, we leveraged a network-suppressive 100 Hz pulsation protocol to balance mechanistic efficacy with thermal safety. While Murphy et al. demonstrated that pulsed ultrasound with a PRF of 900 Hz can selectively activate inhibitory parvalbumin interneurons and lead to a net suppression of pyramidal cells for up to 10 s after stimulation^26^, directly applying such a high PRF to our 10-min therapeutic protocol would pose a significant risk of tissue thermal damage due to insufficient inter-pulse intervals for heat dissipation. To mitigate this, our 100 Hz protocol provides a significantly longer off-time between pulses (9.25 ms vs. <1 ms at 900 Hz), facilitating efficient thermal diffusion. Crucially, this 100 Hz pulsation effectively suppressed PTZ-induced acute epileptiform discharges and was also capable of decreasing kainic acid-induced chronic epileptiform activity up to 7 weeks in our previous studies^23,27^.

Hence, we adopted a low-duty-cycle, low-PRF FUS protocol to minimize skull-base heating while still achieving robust, sustained suppression of PTZ-induced epileptiform activity. This delayed yet lasting suppression is consistent with prior reports of clinical benefit in epilepsy management using noninvasive neuromodulation^57^. These findings highlight that effective clinical application of FUS for epilepsy will require not only low-intensity parameters, but also repeated delivery and prolonged stimulation to ensure both safety and sustained therapeutic benefit for patients with drug-resistant epilepsy.

This study has several limitations that should be addressed in future work. First, the use of an acute seizure model under anesthesia may not fully capture the complexity, variability, and chronic nature of human epilepsy^25^. Our experiments do not indicate the potential duration of any benefits coinciding with sonication-induced calcium changes. Future studies should incorporate chronic epilepsy models in awake, freely moving animals to better approximate clinical conditions. Second, while fiber photometry provides valuable real-time insight into population-level calcium dynamics, it lacks spatial resolution and cell-type specificity. Integration of GCaMP fiber photometry with higher-resolution techniques, such as microendoscopy or two-photon imaging, would allow cell-type-specific analysis of neural activity during FUS neuromodulation. Additionally, investigating long-term synaptic plasticity^58^, the role of nonneuronal elements, such as astrocytes and microglia^17^, and the potential impact of FUS on blood-brain barrier permeability^15,16^ could provide deeper mechanistic insight into FUS-induced neuromodulatory processes. Lastly, we have shown a correlation of FUS, calcium activity and markers of seizures, but we cannot claim that reduced calcium activity is a primary cause of any changes, rather than the consequence of an FUS effect on another factor.

## Methods

### Animals and surgical procedures

A total of 41 male C57BL/6J mice (8–14 weeks old; BioLASCO, Taipei, Taiwan) were housed under a 12-h light/dark cycle with ad libitum access to food and water. The mice were divided into an in vivo cohort (for GCaMP and/or ECoG recordings; *n* = 23) and an ex vivo cohort (for immunofluorescence staining; *n* = 18). All animal procedures were approved by the Institutional Animal Care and Use Committee of National Taiwan University (IACUC nos. NTU-112-EL-00013 and NTU-112-EL-00069) and conducted in accordance with the NIH Guide for the Care and Use of Laboratory Animals.

**Fig. 5:**
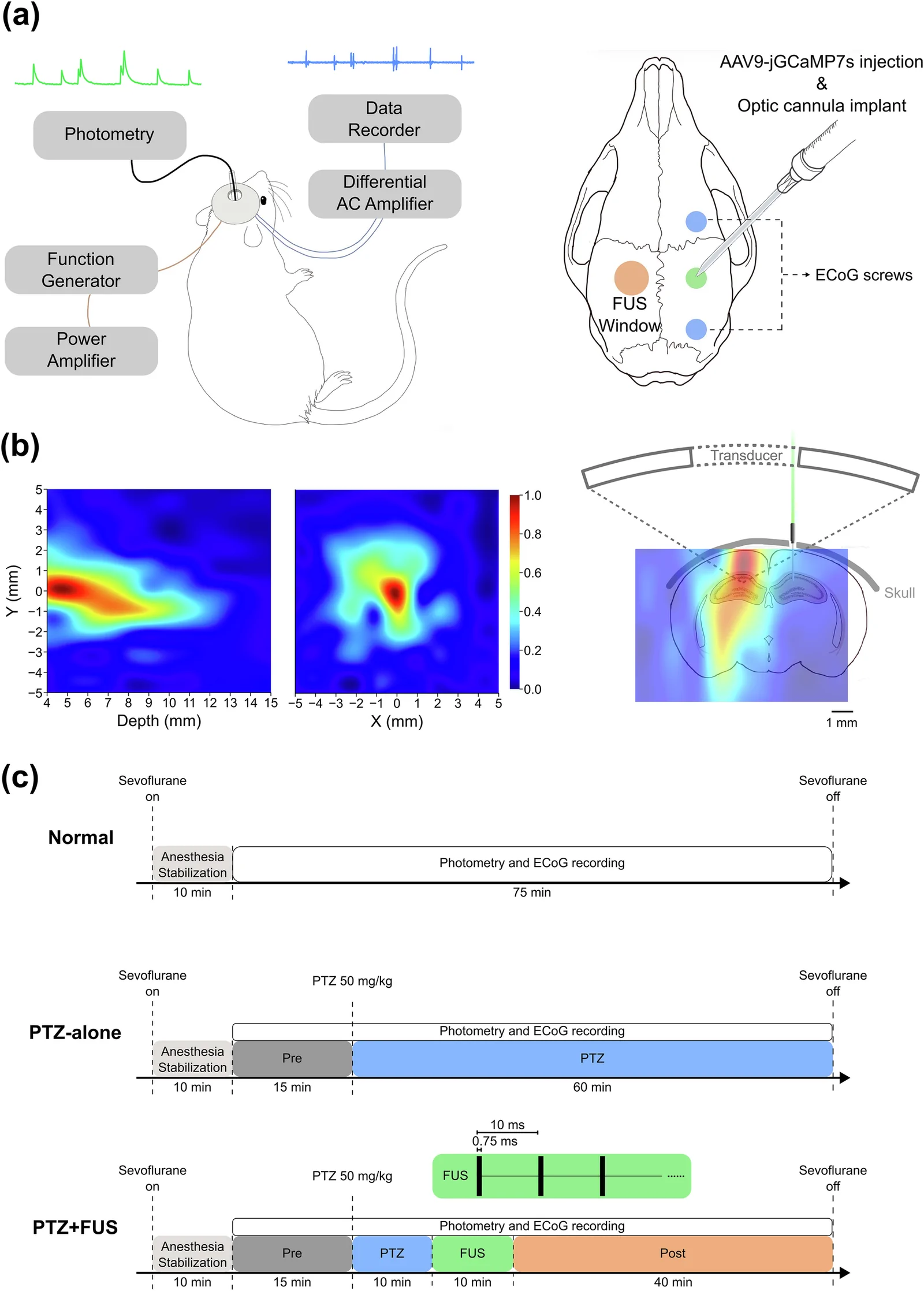
Experimental protocol and ultrasound stimulation parameters for assessing FUS effects on epileptiform activity in mice. **a** Schematic of the simultaneous GCaMP fiber photometry and ECoG setup with AAV-GCaMP7s injection and fiber implantation. **b** Diagram showing ultrasound transducer placement and focal zone dimensions (~2 mm lateral, ~4 mm axial) measured transcranially, with an intensity map overlay. **c** Timeline of the experiments. PTZ + FUS group includes baseline (15 min), PTZ induction (10 min), FUS stimulation (10 min), and post-stimulation recording (40 min); PTZ (50 mg/kg) was used to induce epileptiform activity, and FUS was delivered using 0.75 ms pulses at 10 ms intervals.

A schematic overview of the experimental setup, including viral injection, optical fiber implantation, and ECoG electrode placement, is shown in Fig. 5a. For surgical procedures, mice were anesthetized with 3–5% isoflurane for induction and maintained at 1.5% during the procedure using a stereotaxic frame (RWD Life Science, China). Body temperature was maintained using a thermostatically controlled heating blanket. After a midline scalp incision, the skull surface was exposed and cleaned.

An AAV-GCaMP construct was employed to deliver the gene encoding GCaMP into neurons within the targeted brain region, and GCaMP is expressed to reflect free intracellular calcium dynamics. Stereotaxic burr holes were drilled for viral injection and optic cannula placement. pGP-AAV9-syn-jGCaMP7s-WPRE (5 × 10^12^ vg/ml; 100 nL at 2 nL/s; #104487, Addgene) was unilaterally injected into the CA1 region of the hippocampus (coordinates: AP −2.06 mm, ML −1.30 mm, DV −1.50 mm) using a nanoliter injector (R-480, RWD Life Science, China) connected to a pulled glass micropipette. The micropipette was left in place for 10 min post-injection to minimize backflow. A fiber optic cannula (ferrule size ∅ 1.25 mm, core 400 μm, NA 0.50, length 4 mm; RWD Life Science, China) was implanted just above the injection site.

For ECoG recordings, two skull screws were inserted into the ipsilateral hemisphere at the following coordinates: ECoG1: AP 0 mm, ML −1.3 mm; ECoG2: AP −3.5 mm, ML −1.3 mm. Fiber optic cannula and electrodes were secured using dental acrylic, and a 2 mm diameter area contralateral to the fiber optic cannula was intentionally left clear of dental acrylic to allow FUS exposure. The surface of dental cement was smoothed to reduce irregular refraction and scattering of the acoustic wave. Animals were allowed to recover for at least three weeks prior to experimental recordings to ensure sufficient viral expression.

### Focused ultrasound stimulation setup and parameters

Transcranial FUS was delivered using a single-element 1 MHz transducer with a spherical cap geometry (20 mm diameter, 5 mm depth, and 16 mm radius of curvature). The transducer featured a 6 mm diameter center hole designed to accommodate the GCaMP fiber-optic cannula for concurrent hippocampal recording. The transcranial acoustic field was characterized underwater using a needle hydrophone (TC4038, Teledyne, Denmark) with a piece of mouse cranium positioned between the transducer and the sensor. The resulting transcranial focal beam spans 2 mm laterally and 4 mm axially, as illustrated in Fig. 5b. During the experiment, the transducer was secured in a 3D-printed holder attached to a 3-axis manipulator. A 2 mm standoff distance was maintained between the holder and the skull surface to align the focal zone with the hippocampus.

Ultrasound waveforms were generated using a function generator and amplified prior to transmission. FUS sonication was applied using a pulse repetition frequency of 100 Hz, pulse duration of 0.75 ms, DC of 7.5%, and a total stimulation duration of 10 min. The acoustic pressure was fixed at 0.2 MPa (after considering 20% skull attenuation), which equals to a transcranial mechanical index (MI) of 0.2. The corresponding transcranial FUS intensities, reported in spatial-peak pulse average (*I*_sppa_) and spatial-peak temporal average (*I*_spta_), were 1.62 and 0.16 W/cm^2^, respectively. The parameters were carefully chosen and slightly reduced based on our previous study^23^ to avoid potential tissue heating or damage. Sonication was synchronized with the start of the second recording epoch (immediately after PTZ injection) using a TTL-triggered timing system integrated with both fiber photometry and ECoG acquisition systems.

### Pentylenetetrazol-induced acute seizure model and experimental timeline

To establish an acute seizure model, mice were intraperitoneally injected with PTZ (50 mg/kg B.W.; Merck) to induce generalized seizures. The experimental timeline, illustrated in Fig. 5c, outlines the sequence of PTZ administration, a brief recovery period, FUS stimulation, and continuous electrophysiological recordings. Neural activity was recorded across four distinct phases: a 15-min baseline prior to PTZ injection (Pre), 10 min immediately following PTZ administration (PTZ), 10 min during FUS stimulation (FUS), and a subsequent 40-min post-stimulation phase (post). These time windows allowed for the quantitative assessment of FUS-mediated effects on PTZ-induced epileptiform discharges. To evaluate the effects of FUS, animals in the in vivo cohort (*n* = 23) were subdivided into three experimental groups. In the normal group (*n* = 6), mice underwent a full 75-min recording session without receiving PTZ or FUS. In the PTZ-alone group (*n* = 7), animals received PTZ but did not undergo FUS, resulting in a total recording duration of 75 min. In the PTZ + FUS group (*n* = 10, half of the mice were implanted with optic fiber and electrode, the remaining half were implanted exclusively with fiber), animals were administered PTZ, followed by a single FUS session initiated 10 min after injection, and were subsequently recorded for 40 min following stimulation.

### Calcium signal analysis

Neuronal calcium GCaMP fluorescence signals were acquired using a dual-color fiber photometry system (R810, RWD Life Science, China), using 470 nm excitation for GCaMP-dependent signals and 410 nm for isosbestic control. Both signals were sampled at 100 Hz. To correct for non-biological artifacts, slow baseline drift due to photobleaching was fitted and removed from both channels using an iterative weighted least squares regression. Next, motion artifacts were corrected by regressing the baseline-corrected 410 nm signal onto the 470 nm signal using robust least squares (Fig. S4). The corrected signal was then expressed as Δ*F*/*F* (%) relative to baseline fluorescence levels. Calcium transients were defined as events exceeding two median absolute deviation (MAD) above the baseline noise level. For each time epoch, both the frequency and amplitude of transient events were quantified. In addition, the area under the curve (AUC) was calculated to estimate cumulative calcium load, reflecting overall intracellular calcium activity. Comparisons across seven experimental time periods: Pre, PTZ, FUS, Post10 (0–10 min after FUS), Post20 (10–20 min), Post30 (20–30 min), and Post40 (30–40 min) were performed to evaluate changes in calcium dynamics and the extent of FUS-mediated suppression of PTZ-induced calcium waves.

### Electrocorticography acquisition and spectral analysis

ECoG signals were acquired via skull screws implanted in the cortical surface, amplified by a differential AC amplifier (Model 1700, A-M Systems, USA), and digitized at a sampling rate of 200 Hz by a data recorder (IX-214, iWorx Systems, USA). Signals were band-pass filtered between 0.5 and 100 Hz. Time-frequency decomposition was performed on 10-min periods using the short-time Fourier transform with 5-s Tukey windows, 12.5% overlap, and an FFT length of 8192 points. PSD analysis was conducted using the fast Fourier transform. Spectral power was quantified within standard EEG frequency bands: delta (1–4 Hz), theta (4–8 Hz), alpha (8–13 Hz), beta (13–30 Hz), and gamma (30–60 Hz), for each defined time period. High-amplitude spike discharges were identified as events exceeding three times the MAD of the baseline signal, and the amount of discharge was computed per 10-min periods for comparison across groups.

### Signals synchrony analysis

To evaluate the temporal synchrony relationship between neuronal electrical and calcium activity, we conducted a cross-correlation analysis between ECoG and GCaMP fiber photometry signals. ECoG signals were first down-sampled to 100 Hz to match the photometry sampling rate. The time axes of both signals were realigned so that the time of PTZ injection was set to zero. Three distinct periods were analyzed: Pre, PTZ, and Post40. For each period, the ECoG and GCaMP fiber photometry signals were low-pass filtered below 2 Hz using an 8th-order Butterworth filter to extract only slow-oscillating burst components. The normalized cross-correlation between ECoG and photometry traces and the autocorrelation of individual signals were computed, and the time lag of the cross-correlation was identified to determine the temporal synchrony between electrophysiological and calcium signals.

### Histological and immunofluorescent analysis

To preclude the endogenous fluorescence crosstalk and bleed-through across the broad spectrum during the staining and quantification processes, the ex vivo cohort (*n* = 18) did not receive AAV-GCaMP transfection. Upon completion of the experimental procedures, mice were deeply anesthetized with xylazine (10 mg/kg B.W., i.p.) and transcardially perfused with phosphate-buffered saline, followed by 4% paraformaldehyde (PFA). Brains were post-fixed overnight in PFA and subsequently cryoprotected in 30% sucrose before embedding in OCT compound. Coronal brain sections (50 μm) were obtained using a cryostat and processed for free-floating immunofluorescence. Sections were blocked with 10% normal goat serum and 0.5% Triton X-100 for 2 h at room temperature, then incubated overnight at 4 °C with the following primary antibodies: rabbit anti-NMDAR2B (1:500, ab65783, Abcam), rabbit anti-GAD65 + 67 (1:250, ab183999, Abcam) and guinea pig anti-c-Fos (1:500, 226 308, Synaptic Systems). On the following day, sections were incubated with Alexa Fluor 647-conjugated goat anti-rabbit (1:500) and Alexa Fluor 405-conjugated goat anti-guinea pig (1:250) secondary antibodies. Stained sections were mounted using Fluoromount-G with DAPI and imaged using either a Zeiss LSM 780 confocal microscope or a Leica Mica widefield microscope. Image processing and analysis were performed using Zen (Zeiss) and LAS X (Leica) software. Quantification was performed by calculating the percentage of signal-positive area within researcher-defined regions of interest in the DG.

### Statistical analysis

All data were analyzed using MATLAB, Python and R. Quantitative results are presented as mean ± standard error of the mean.

For longitudinal data, the normality of model residuals was assessed by the Shapiro–Wilk test. Longitudinal trends between groups were analyzed using linear mixed models (LMM) and analysis of variance with Satterthwaite’s approximation for degrees of freedom. The post hoc comparisons at specific time points were performed using estimated marginal means. For ECoG band power analysis, data were normalized to baseline. Statistical comparisons involving the baseline were performed using two-tailed one-sample t-test. Comparisons between post-baseline time points were performed using two-tailed paired t-test. For immunofluorescence quantification, the nonparametric Wilcoxon rank-sum test was employed.

Holm–Bonferroni correction was applied to control for multiple comparisons within each group of post hoc. Statistical significances were defined as *p* < 0.05, *p* < 0.01, and *p* < 0.001. The significances from LMM, one-sample t-test, paired t-test and Wilcoxon rank-sum test were indicated by ^★^, and the significances from post hoc comparisons were marked by ^#^. Sample sizes for each group and experimental condition are provided in the corresponding figure legends.

## Supplementary information

## Data Availability

The datasets generated and analyzed during the current study are not publicly available due to ongoing commercial development and intellectual property constraints related to specific therapeutic ultrasound parameters. However, the data are available from the corresponding author upon reasonable request.
