# Supplementary material for: GCaMP fiber photometry reveals mechanistic insights into focused ultrasound neuromodulation in acute seizures
Source: NPJ Acoust. 2026 Sep 2;2(1):35. doi: 10.1038/s44384-026-00057-6 (PMC13538028; doi:10.1038/s44384-026-00057-6)
Supplement: Supplementary file 1 [file 44384_2026_57_MOESM1_ESM.pdf]

Supplementary Materials

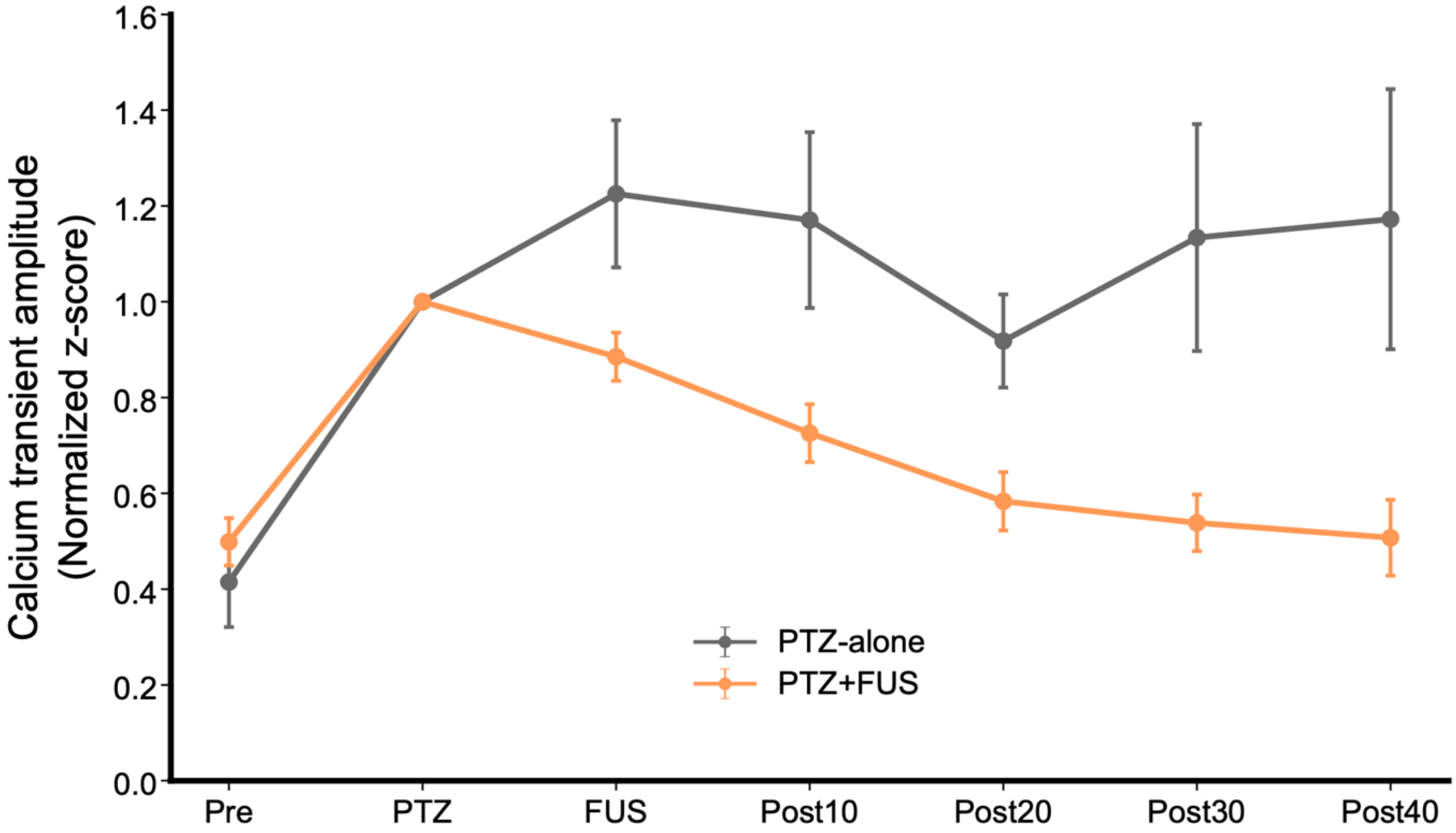

Fig. S1. Focused ultrasound attenuates PTZ-induced calcium transient amplitudes.

Time course of calcium transient amplitude (calculated as peak prominence) across each period. Data are standardized as z-scores and normalized to the PTZ period (set to 1.0). While The PTZ+FUS group (n = 10) exhibits a sustained reduction trend in transient amplitude following FUS compared to the PTZ-alone control group (n = 7), statistical comparisons using a linear mixed model reveals no significant difference between groups ($F_{(6, 88.06)} = 2.15$, $p = 0.0555$). Data are shown as mean ± SEM.

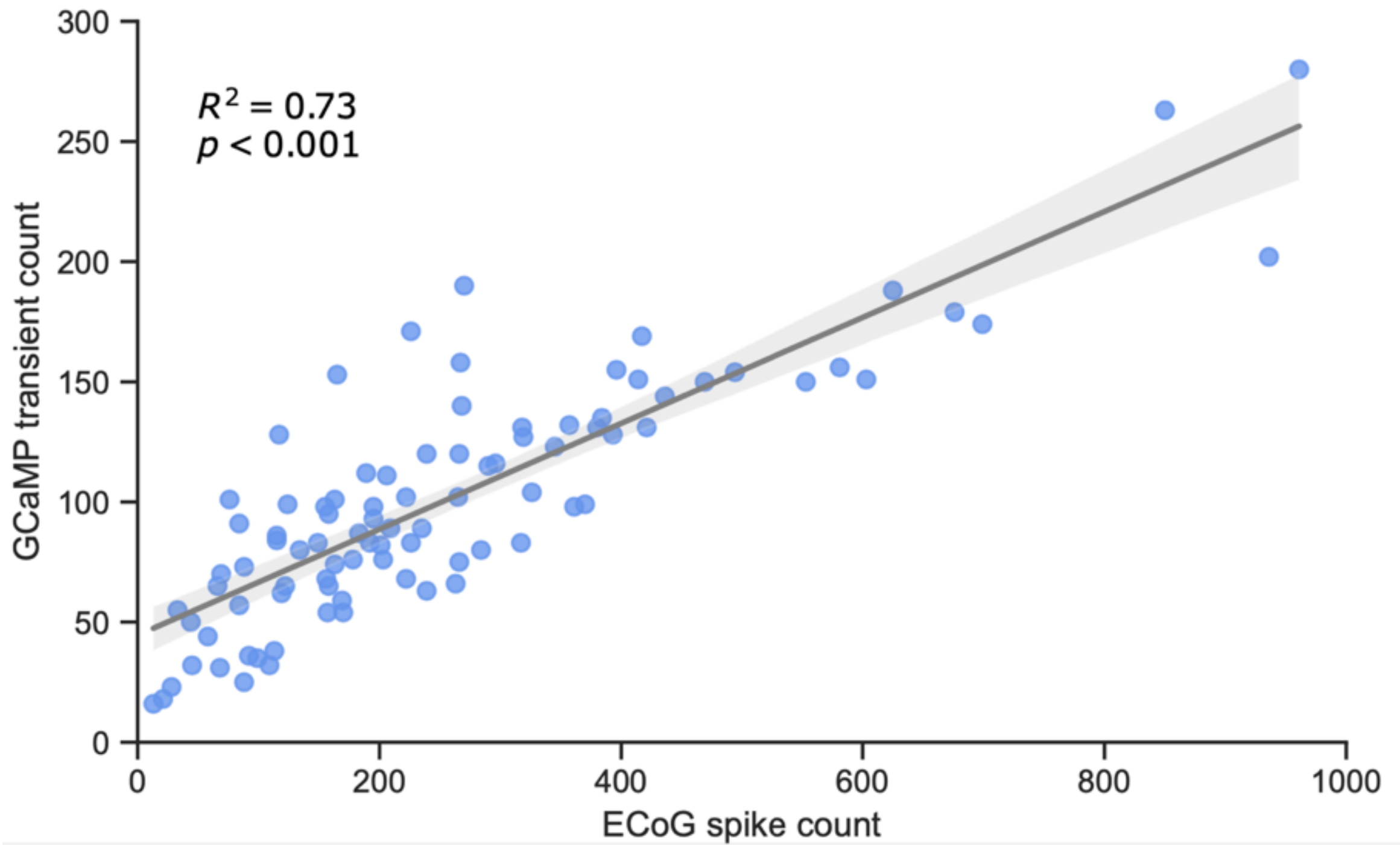

Fig. S2. Correlation between ECoG spike counts and GCaMP transient counts. The scatter plot displays 77 data pairs from 7 periods across 11 trials (n = 7). Linear regression analysis (R^2 = 0.73, solid line) showing the relationship between ECoG spike counts and GCaMP transient counts. The gray shading spans the 95% confidence interval. Statistical significance was computed using a two-tailed Spearman correlation ($p < 0.001$).

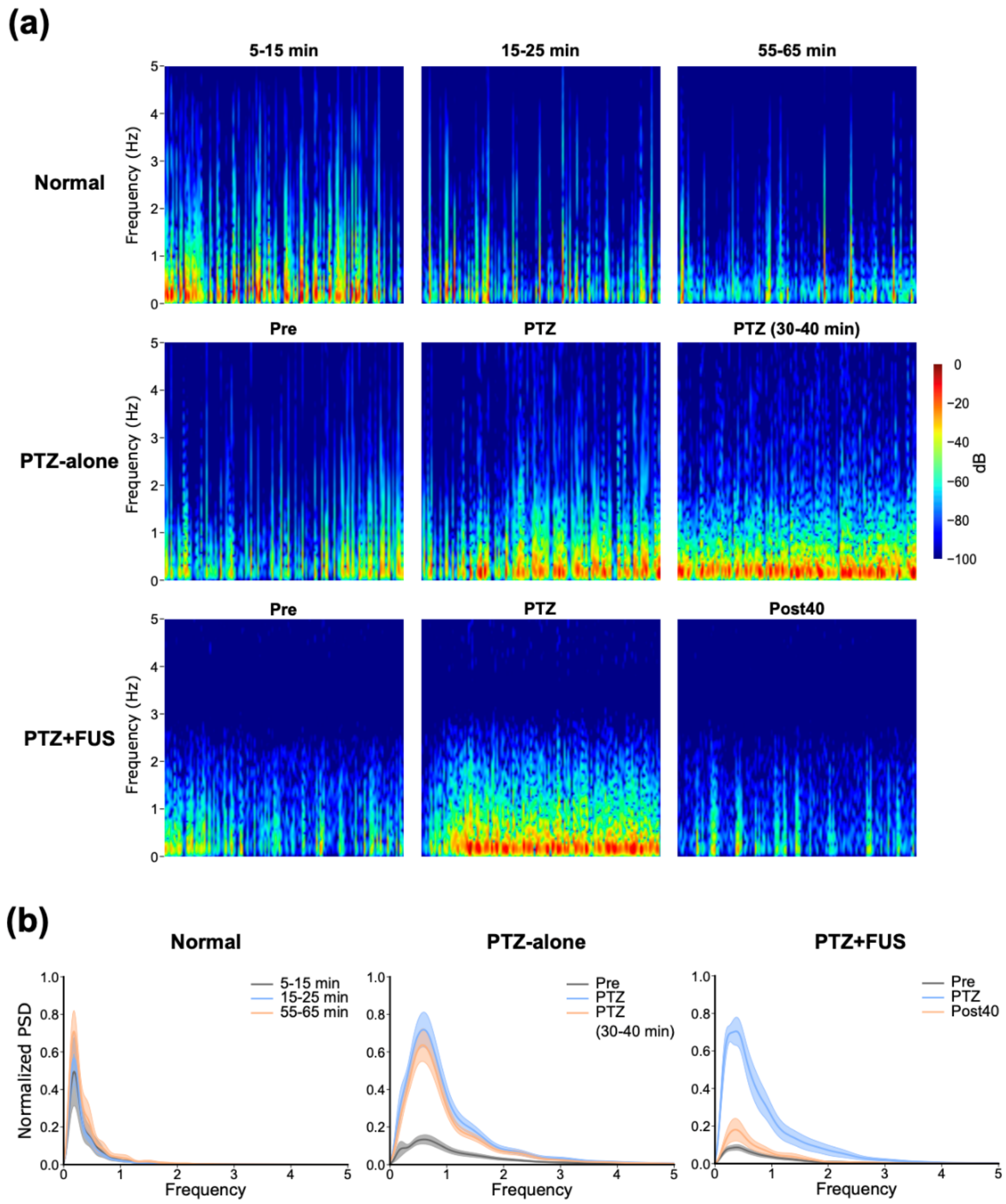

Fig. S3. Spectral analysis of GCaMP signal demonstrates attenuation of epileptic activity by FUS. **(a)** Representative spectrograms of GCaMP calcium signals across three experimental phases: baseline (Pre), during PTZ-induced seizures (PTZ), and post-intervention (Post40: 30–40 minutes after PTZ). PTZ induced a marked increase in delta band (1–4 Hz) spectral power. In contrast, FUS-treated mice (bottom row) exhibited reduced low-frequency power following sonication. **(b)** Normalized PSD plots for Normal, PTZ-alone, and PTZ+FUS groups. Data are presented as mean ± SEM.

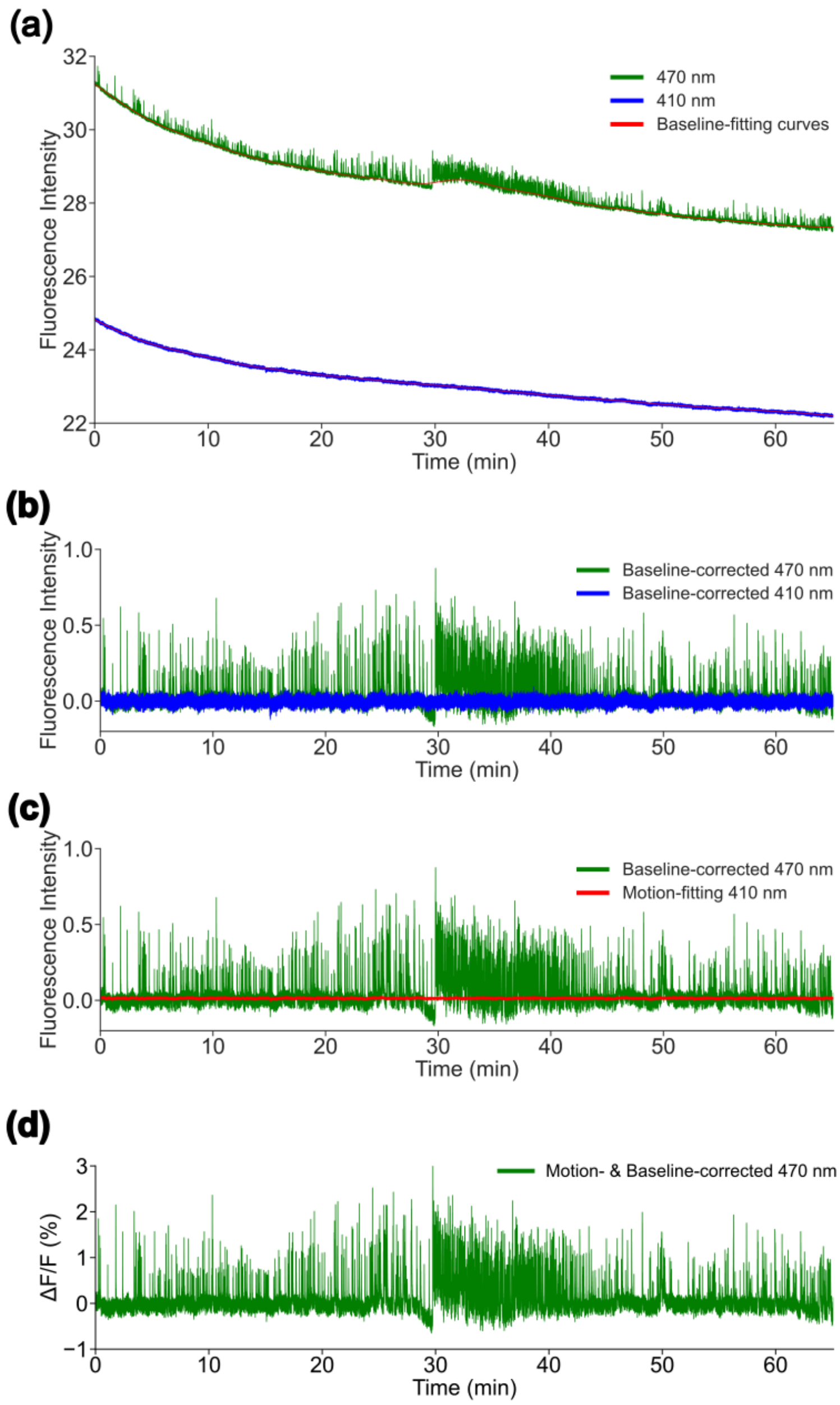

Fig. S4. Representative workflow for fluorescence signal correction. (a, b) Raw fluorescence signals for 470 nm (green) and 410 nm (blue) channels exhibit decay due to photobleaching. This drift was corrected by fitting an iterative weighted least square regression (red lines in **a**) to obtain the detrended baselines shown in **(b)**. **(c, d)** To remove motion artifacts, the detrended 410 nm signal was fitted to the 470 nm signal using robust least square regression (red line in **c**). The final motion-corrected trace (**d**) is expressed as percentage change in fluorescence ($\Delta F/F$).
